# Observational Diagnostics: The Building Block of AI-Powered Visual Aid for Dental Practitioners

**DOI:** 10.3390/bioengineering12010009

**Published:** 2024-12-25

**Authors:** Ruchika Raj, Ravikumar Rajappa, Vijayalakshmi Murthy, Mahyar Osanlouy, Daniel Lawrence, Mahen Ganhewa, Nicola Cirillo

**Affiliations:** 1 CoTreat, CoTreat Pty Ltd., Melbourne, VIC 3000, Australiadaniel.lawrence@cotreat.com.au (D.L.); max.ganhewa@cotreat.com.au (M.G.); 2 Faculty of Medicine, Dentistry and Health Sciences, The University of Melbourne, 720, Swanston Street, Carlton, VIC 3053, Australia

**Keywords:** artificial intelligence, observational diagnostics, OD-based algorithms, evidence-based treatment, dentistry, AI applications, AI-powered visual aid

## Abstract

Artificial intelligence (AI) has gained significant traction in medical image analysis, including dentistry, aiding clinicians in making timely and accurate diagnoses. Radiographs, such as orthopantomograms (OPGs) and intraoral radiographs, along with clinical photographs, are the primary imaging modalities employed for AI-powered analysis in the dental field. In this review, we discuss the most recent research and product developments concerning the clinical application of AI as a visual aid in dentistry and introduce the concept of Observational Diagnostics (ODs) as a structured method to standardise image analysis. ODs serve as foundational elements for AI-driven diagnostic aids and have the potential to improve the consistency and reliability of diagnostic data used in treatment planning. We provide illustrative examples to demonstrate how ODs not only represent a significant advancement towards more precise diagnostic aids but also provide the basis for the generation of evidence-based treatment recommendations. These OD-based algorithms have been integrated into chairside AI applications to streamline clinical workflows to improve consistency, accuracy, and efficiency.

## 1. Introduction

Artificial intelligence (AI) is rapidly transforming the fields of medicine and dentistry, offering significant potential to enhance diagnostic accuracy, optimise treatment outcomes and in doing so, improve patient care [1]. In dentistry, AI is being leveraged to aid clinicians in the detection of oral and dental conditions, with the aim of improving the accuracy of radiographic assessments and facilitating more efficient patient management. Specifically, a variety of machine learning (ML) and deep learning (DL) techniques for dental image analysis are being evaluated to assist clinicians in making definitive diagnoses and choosing the appropriate intervention promptly for better patient outcomes. In one example, a recent scoping review has shown that AI-based image analysis was applied to virtually all fields of dentistry, with oral medicine (19%), oral and maxillofacial surgery (16%), and operative dentistry (15%) taking the lead [2]. Imaging modalities mostly used for AI analysis in dentistry are radiographs (orthopantomograms (OPGs) and intra-oral radiographs) and clinical photographs.

The application of AI in dental imaging is currently focused on the automation of image interpretation to assist practitioners in identifying pathologies and treatment outcomes. However, despite these advancements, there is a lack of standardisation in the way AI algorithms interpret imaging data. In this article, we aim to review the common image modalities used in dentistry and to showcase how the development of discrete diagnostic observations constitutes the building blocks of AI-powered visual aids for practitioners. We further discuss how these Observational Diagnostics (ODs) can serve as a structured framework that bridges the gap between AI technology and clinical applicability, ensuring that AI-generated diagnostic data are both reliable and actionable.

## 2. Artificial Intelligence for Radiographic Images

Human readers are limited in their ability to assess numerous qualitative features when interpreting medical images, and their performance can vary. AI, however, can analyse a vast array of quantitative features consistently and reproducibly. These imaging biomarkers can be used for prediction and risk assessment, diagnosis, prognosis, and treatment response. The importance of integration of AI into radiology to reduce human errors, increase efficiency, and achieve objectives with minimal manual input is now widely appreciated [3]. For example, traditional diagnostic methods, such as X-rays, may miss early signs of disease that are not yet visible to the naked eye [4]. AI systems may be capable of systematically detecting these subtle changes by analysing radiographs and imaging modalities alone or in combination, apparently with remarkable precision [5], thus allowing timely management [6]. To exploit this potential, an AI-based mammography tool has been developed by Google Health and has shown a 9.4% reduction in false negatives and a 5.7% reduction in false positives [4]. Specifically, in the US dataset, the AI system showed a sensitivity of 94.5% and a specificity of 91.1%, compared to the average sensitivity of 90.5% and specificity of 88.0% achieved by human radiologists.

**Figure 1:**
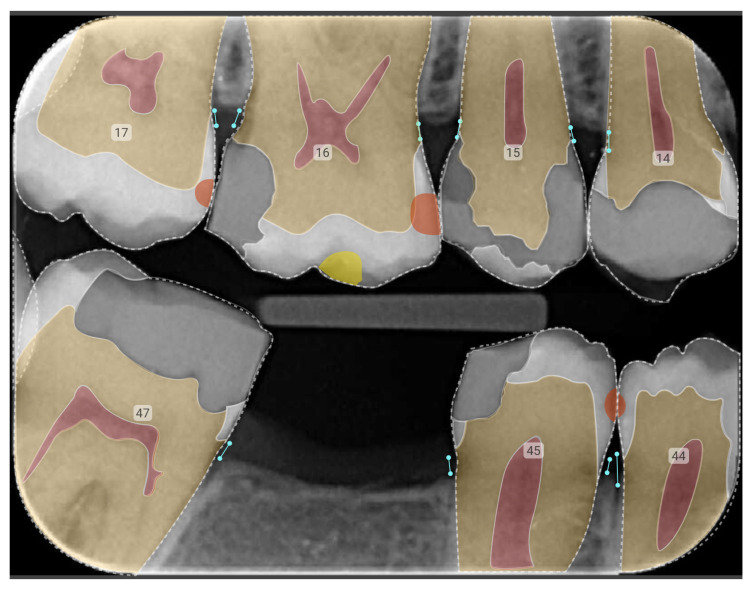
Example of annotated dental X-rays highlighting bone loss (turquoise lines) and radiolucency (orange semicircles). The numbers indicate the tooth numbers based on the FDI notation system.

In dentistry, AI tools for interpreting dental radiographs leverage ML and DL algorithms to analyse images such as OPGs and intraoral radiographs. The models most commonly used in diagnostic assistance systems are Convolutional neural networks (CNNs), a type of deep learning model particularly well-suited for analysing visual data. A review of 36 studies found that CNNs were most often used in general dentistry [7]. These technologies enable the detection of various dental conditions, including caries, periodontal diseases, and other abnormalities, by recognising image patterns or simply by categorising these patterns accurately and in a time-efficient manner to aid a real-time diagnosis (Figure 1). Early data show that the integration of AI in radiographic analysis has the potential to not only enhance diagnostic accuracy, but also streamline workflow, allowing practitioners to focus on patient care rather than time-consuming image evaluations [2].

A common application of AI in dentistry is for the detection of caries, and a CNN-based tool was found to detect caries in radiographs with an accuracy comparable to human examiners, achieving an area under the curve (AUC) of 0.89 [7]. In another example, a study by Lee et al. (2018) [8]. evaluated the efficacy of a deep learning-based CNN algorithm for the detection and diagnosis of dental caries on periapical radiographs using 3000 periapical radiographic images. In this study, the deep CNN algorithm exhibited strong performance in accurately detecting dental caries on periapical radiographs. Specifically, the diagnostic accuracies of premolar, molar, and both premolar and molar models were 89.0% (80.4–93.3), 88.0% (79.2–93.1), and 82.0% (75.5–87.1), respectively. The deep CNN algorithm achieved an AUC of 0.917 (95% CI 0.860–0.975) on premolar models. This early study highlighted that the diagnostic accuracy of CNNs in dentistry could approach the level of human expertise.

As medical decision-making can be noisy, particularly with the interpretation of imaging [3], AI has the potential to achieve greater consistency and accuracy. For example, a randomised controlled trial evaluating the accuracy of AI-based software with human interpretation in the diagnosis of dental caries by using intra-oral radiographs showed that AI-based software achieved a sensitivity of 88%, a specificity of 91%, and an overall accuracy of 89%, surpassing human examiners, who showed sensitivity, specificity, and accuracy rates of 84%, 88%, and 86%, respectively. In this regard, the term “computer-assisted diagnosis” has been coined to refer to the increasing importance of AI in reducing human diagnostic errors as technology advances. [9] Published research reports assessing AI-assisted tools in diagnosing dental pathologies by analysing dental radiographs and clinical photographs are shown in Table 1.

Notably, many of these advances have translated into diagnostic aid tools. Examples of AI-powered software currently used chairside in dental practice, as accessed on 30 September 2024, include CoTreat (https://www.cotreat.ai) DentrixDetect (VideaHealth) (https://www.videa.ai/practices), and Second Opinion (Pearl) (https://www.hellopearl.com/products/second-opinion); these and others are discussed in Section 6.

In summary, the current literature suggests that AI systems have remarkable capabilities in clinically relevant image segmentation and classification that are comparable to, if not better than, human performance [18]. These findings underscore AI’s potential to improve diagnostic accuracy, ensure consistency, and reduce variability in dental radiographic assessments [17] and demonstrate a paradigm change brought about by the introduction of artificial intelligence into diagnostic imaging [18,19].

## 3. Artificial Intelligence for Clinical Images

The diagnostic process in dentistry can sometimes be subjective, with clinicians relying on their own experience and expertise to interpret clinical images. This subjectivity can lead to variability in diagnoses, especially in more complex cases. Hence, analysis of clinical images can help the clinician detect dental disease more consistently, improve diagnostic accuracy, and personalise treatment plans. The integration of AI in clinical image interpretation is still in its early stages, but its potential is vast. Continuous learning from large datasets allows AI to stay current with the latest research and clinical guidelines [23]. One of the key capabilities that have been leveraged to integrate AI into image analysis is the detection of changes in colours and patterns, which have natural applications in the analysis of tooth discolouration, decay, and mucosal abnormalities.

The prototype of AI-powered image analysis tool in dentistry is again well versed for caries detection. For decades, dentists have relied on a combination of visual–tactile (including probing) and radiographic examination to identify dental caries accurately [24]. Probing involves the use of dental instruments to feel for soft spots or cavities in the teeth. Radiographs provide a more detailed view of tooth structure, allowing dentists to visualise hidden caries beneath the enamel. While this approach has served dentistry well, it is not without its limitations and drawbacks. Probing can sometimes be uncomfortable for patients and may even damage weak teeth. Additionally, radiographs expose patients to ionising radiation, albeit at low levels, which can accumulate over time and potentially pose health risks [24]. Studies comparing the sensitivity of visual examination to radiographs for detection of tooth decay have yielded intriguing results. In recent systematic reviews, visual inspection has been found to have a higher sensitivity (i.e., the proportion of decayed teeth correctly identified by the test) in detecting early caries and enamel lesions, especially non-cavitated lesions that may be missed by traditional radiographs [5,6]. Even more surprising were the findings that visual inspection performed better than X-rays in proximal surfaces of permanent teeth, too. By catching caries at their earliest stages, dentists can implement preventive measures, such as remineralization therapies, to arrest caries’ progression before they become problematic. Therefore, AI can leverage the accuracy and safety of visual inspection for the detection of caries [25].

In addition to hard tissues, AI has shown potential in detecting a wider range of oral diseases. For example, changes in the colour and texture of the oral mucosa have been used to train machine learning algorithms to recognise oral mucosal pathology, particularly oral cancer and potentially malignant disorders, which remain a significant public health issue worldwide [26]. Additionally, AI models can assist in analysing microscopic slides of oral tissue, reducing the burden on pathologists. For example, AI can aid in detecting oral squamous cell carcinoma (OSCC) from histopathological images with remarkable accuracy [27]. Furthermore, in regions with limited access to specialists, AI-based tools can be incorporated into telemedicine platforms enabling remote diagnosis. Clinicians can capture images of suspicious lesions, which are then analysed by AI models trained to detect oral cancer. This approach facilitates timely referrals and reduces diagnostic delays [28].

In summary, by leveraging machine learning algorithms, AI can assess a wide range of dental conditions, including but not limited to caries. Current evidence suggests that AI-assisted detection has the potential to significantly improve diagnostic accuracy while reducing the likelihood of misdiagnosis or missed lesions. This technological advancement is poised to enhance both diagnostic precision and patient outcomes in dental care.

## 4. The Concept of Observational Diagnostics (ODs) and Its Use for Diagnosis and Treatment Planning

Unsupervised, semi-supervised, and supervised learning are the three broad categories in which ML is classified [18], and this distinction has salient clinical implications. Briefly, “supervised learning” refers to algorithms that work with labelled training data, where clinical annotations, typically provided by radiologists or clinicians, guide the system. As this approach works entirely with labelled data, where the inputs (e.g., medical images) are paired with corresponding outputs (e.g., diagnoses), it aims to reproduce human decisions and learns to predict outcomes based on these labelled examples. As the system allows the prediction of specific pathologies or anomalies with high accuracy, it has immediate clinical applications [29,30]. Conversely, in the unsupervised approach, large volumes of imaging data are analysed without any specific information. By analysing pixel intensity, texture, or shape, these algorithms explore the data to uncover hidden patterns or clusters of information that may indicate a diagnostic finding. This can be particularly useful for population-wide studies or rare disease detection. However, the system may miss clinically relevant details since it lacks direct guidance from medical experts [31,32]. Semi-supervised methods are particularly useful when there is a mixture of labelled (annotated) and unlabelled data. In dentistry, this approach can be applied when a portion of the dataset has clinical annotations, while the rest is unannotated. The system is trained in the small pool of labelled data, learning clinically relevant features, and can generalise its knowledge to the unlabelled portion. In medical imaging, this could be beneficial in cases where clinicians annotate a limited number of images, allowing the system to extrapolate and assist in diagnostics for larger datasets [33,34].

### 4.1. Observational Diagnostics for Diagnosis

The supervised approach is the one that more closely reproduces the clinician’s diagnostic cues and, to some extent, can be considered as a hybrid (human and AI) workflow. To enable this, however, diagnostic findings must be categorised unequivocally to allow accuracy and consistency. Unfortunately, many dental diseases have different and often non-standardised diagnostic criteria reported in the literature. In order not to minimise variation and smoothen the diagnostic workflow, the standardisation of diagnostic features for ML has been applied to several dental AI-powered software, such as CoTreat’s ODs. ODs have been developed as a structured approach to categorise and interpret diagnostic observations drawn exclusively from visual sources, such as radiographs or photographs. ODs can be classified into macro-categories encompassing the broader disease category (e.g., Dental Caries) and subcategories featuring the basic visual characteristics (e.g., Radiolucency), enabling practitioners to utilise these observations as a foundation for AI-driven diagnostic aids in clinical settings [35]. As an illustrative example, the description of ODs for dental caries developed using the existing literature [36,37] is provided in Supplementary Table S1.

In summary, ODs generated by supervised learning provide the basis for diagnostic aid tools, ensuring that image analysis aligns unequivocally with clinical findings. This approach integrates human expertise with advanced computational models, ensuring high precision in detecting abnormalities that may otherwise go unnoticed to the clinician.

### 4.2. Observational Diagnostics for Treatment Planning

While there has been an explosion of research on AI-assisted diagnostics, the utilization of AI for treatment recommendations has received less attention (Table 2), despite research showing that dentists often make clinical judgments intuitively on limited heuristics leading to non-evidence-based treatment decisions [38]. Importantly, research shows that trusted peers’ opinions can lead to modifications in diagnosis and treatment planning by dentists [39]. Hence, it is crucial to maintain a foundation of evidence-based practice when developing and recommending clinical treatments.

Evidence-based clinical guidelines are the result of a collaborative process designed to ensure that recommendations are grounded in current, verifiable evidence while remaining practical, measurable, and achievable. This process ensures that the guidelines are tailored to each dental pathology, as defined by the diagnostic criteria within the panel’s expertise. As a result, clinicians gain streamlined access to concise, clinically relevant evidence that can be integrated into patient care [43]. Similarly, AI-generated treatment guidelines must be accurate, reliable, clinically relevant, and easily translatable into patient care practises. To achieve this, AI systems must be trained to provide treatment plans in accordance with identifiable and classifiable diagnostic patterns. By systematically categorizing dental conditions into specialized fields and labelling them with ODs using standardized terminologies, AI systems can be trained to diagnose dental pathology and recommend appropriate treatment options (Supplementary Table S2). In this regard, we have shown recently that AI can analyse appropriate diagnostic input to provide dentists with patient-specific, evidence-based treatment recommendations or alternatives with high accuracy [44].

While AI performs better than a human in harnessing the available treatment category in a short period of time, identifying the suitable procedures specific to the case that can inform clinical decision-making is an entirely different challenge. Furthermore, in determining the best course of action for an identified abnormality, it is critical to also consider contraindications—situations where specific treatments or procedures could be harmful, ineffective, or inappropriate for certain patients. Both absolute and relative contraindications must be carefully considered when developing treatment guidelines in dentistry to ensure patient safety and optimal outcomes. Therefore, when constructing AI models, it is vital to link each OD and its corresponding treatments or procedures to relevant contraindications. This approach can enhance the AI’s ability to provide tailored, patient-specific recommendations, accounting for both the pathology and the unique medical profile of the patient. An illustrative example of OD-based treatment guidelines is provided in Supplementary Table S2 [36]. Crucially, this level of AI support can be delivered in real-time during patient consultations, enabling dentists to make informed, on-the-spot decisions. Ultimately, the integration of AI into dental practice creates a synergistic relationship between technology and healthcare, enhancing both the decision-making process and, prospectively, the overall quality of patient care [45].

## 5. Case Study: AI-Powered Dental Caries Detection and Management

**Figure 2:**
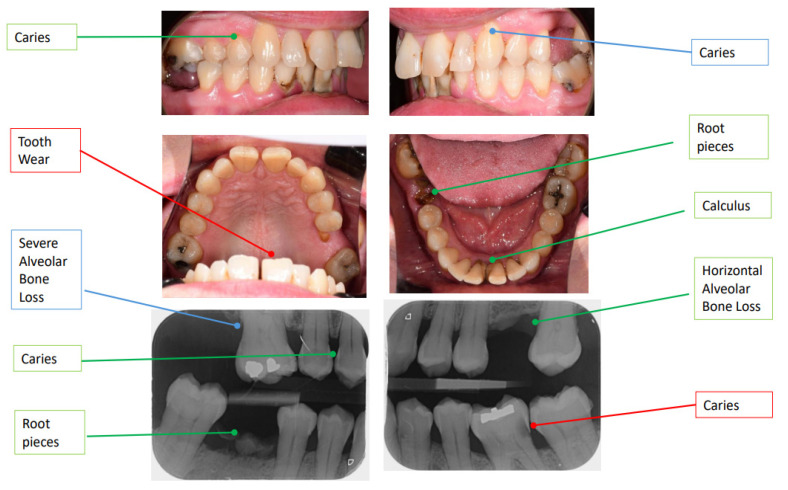
Diagnostic findings using an AI-powered tool. The observations provided by CoTreat Navigator^®^ that match the findings identified by the dentist are marked in green. The findings missed by the dentist (false negative) are marked in red. The dentist’s false positives are marked in blue.

To date, the imaging modality that has been more extensively studied in the context of AI-assisted diagnostic decisions in dentistry are clinical photographs and radiographs for the detection of carious lesions [46]. In this illustrative example, a 55-year-old female was examined by her dentist and diagnosed with moderate gingivitis; treatment was planned accordingly (Supplementary Table S3). Examination of the image dataset provided (intraoral photographs and posterior bitewing (PBW) radiographs) was also conducted by an AI-powered tool (CoTreat’s Navigator^®^) [47] and the output was validated by three authors (R.R., V.M. and R.K.) and subsequently confirmed by the operating clinician (Figure 2). Table S3 shows the complete set of observations and treatments suggested by the operating clinician and Navigator^®^ for this case, whereas the software interface highlighting these findings is provided in Supplementary Figure S1.

For simplicity, here we exemplify the aid that the AI-powered tool provided in facilitating the diagnosis and treatment plan of dental caries, as well as the resulting impact on revenue in Table 3. Navigator^®^ used the pre-specified criteria to determine the presence and severity of caries, generating a corresponding treatment plan based on the guidelines from the International Caries Classification and Management System (ICCMS) and caries classification based on the International Caries Detection and Assessment System (ICDAS). While there was an overall agreement for most observations (true positives), there were discrepancies in a number of findings between the dentist and Navigator^®^ potentially leading to false positives (signalling potential patient harm) and false negatives (signalling potential missed treatment and revenue opportunity) as illustrated in Supplementary Figure S1.

For example, the dentist diagnosed initial caries on the anterior tooth (tooth 23) based on clinical examination and image analysis, recommending adhesive restoration (procedure code 522, cost $228.99) whereas Navigator^®^ did not detect any caries at this site, suggesting a false positive diagnosis leading to unnecessary treatment and an avoidable cost of $228.99 to the patient. Similarly for the same case (tooth 36) the AI-powered tool detected initial-stage caries (ICCMS RA 3) through PBW on the distal surface and moderate caries (ICDAS code 3) via image analysis on the buccal surface of the tooth, which were apparently missed by the dentist, resulting in a false negative diagnosis (Figure 2).

In summary, this case exemplifies the possible benefits of integrating AI technologies into routine dental practice, particularly in enhancing diagnostic and treatment precision and reducing unnecessary procedures and treatment costs for patients. In particular, it showcases the potential of AI-powered tools in aiding the detection of early-stage caries (e.g., tooth 36). Additionally, the false positive finding in the conventional method (tooth 23) highlighted the potential for unnecessary procedures when relying solely on visual and radiographic assessments. The cost implications were also significant, with the AI system helping to avoid unnecessary treatment costs for the patient while maximising revenue opportunities for the dentist. Overall, the integration of AI into dental diagnostics can enhance detection, reduce diagnostic errors, and promote more cost-effective care.

## 6. Discussion

In the last few years, ML has been used in the most disparate fields, from geoscience [48] to chemistry and drug development [49] to healthcare. In medicine, ML models are designed to replicate human knowledge and behaviour to facilitate clinical decision-making. Furthermore, considering AI’s ability to identify changes in pixel brightness and colour beyond that of the human eyes, it can also support practitioners in identifying abnormalities that may not be immediately apparent. AI-based tools have been developed to analyse dental radiographs and clinical images to detect dental conditions such as dental caries and periodontal disease by recognising abnormal patterns. In this regard, we recently developed ML algorithms using ODs as data to train the AI system to categorise and interpret the diagnoses drawn from visual sources like radiographs and photographs. These ODs were structured into macro- and sub-categories which enabled supervised ML to return an output in a form that was clinically meaningful, enabling practitioners to utilise it as a foundation for AI-driven diagnostic aids. Hence, the goal of ODs is to support clinicians by highlighting abnormalities or significant findings that may not be immediately apparent [29,30], and to use these ODs to propose evidence-based treatment plans.

AI can meaningfully assist clinicians with treatment planning, and studies published in this regard support AI’s ability to help in clinical decision-making (Table 2). In traditional settings, clinicians rely on evidence-based guidelines to inform their decisions, ensuring that treatments are grounded in verifiable, current data. These guidelines, developed through a collaborative and transparent process, prioritise practical, measurable, and achievable outcomes [43]. This necessitates the need to train the AI systems with evidence-based, established, treatment guidelines that correlate to the diagnostic information input. ODs are essential in this process, as they provide a standardised framework for categorising dental pathologies. By labelling conditions with specific diagnostic terms, AI can systematically identify dental abnormalities and suggest appropriate treatment options. This process mirrors the evidence-based approaches used by human practitioners but with enhanced speed and precision. Further, it is essential to associate the assigned treatment procedures with known contraindications to prevent any harmful, ineffective, or inappropriate outcomes. This approach enhances the AI’s ability to provide patient-centric recommendations which can not only optimise clinical outcomes but also improve patient satisfaction by reducing unnecessary interventions and treatment costs while ensuring patient well-being.

Building on the encouraging results from recent research, start-up companies have begun to introduce AI-powered products with advanced capabilities, such as diagnosis and treatment planning (Appendix A, Table A1). These systems, when applied to dental radiographs or photographs, assist dentists by automatically generating diagnoses and treatment plans through computer vision technology. In addition to diagnoses and treatment suggestions, some of these products can now, or in the near future, assist the dentist in anatomy annotation, analysing tooth margin for crown preparation, prosthesis design, smile designing, patient data analysis, and monitoring orthodontic treatment outcomes. Currently, however, these plans are limited to relatively simple procedures, like dental restorations, leaving significant room for future enhancement.

The case study on AI-powered dental caries detection underscores the value of AI in improving diagnostic accuracy and treatment planning, streamlining the management of dental conditions. In this instance, the patient image analysis report generated by the AI-powered software allowed early detection and possibly intervention. This improvement in early-stage detection can be attributed to the AI system’s ability to process detailed image data, making it possible to detect subtle indicators of caries that may be missed during a visual examination. The study also highlights the potential risks associated with both AI and traditional methods. The AI system avoided unnecessary treatment costs by identifying a false positive diagnosis from the dentist, showcasing the financial and health-related benefits of accurate diagnostics. On the other hand, AI is not infallible and must be continuously monitored to reassess false negatives, as observed in the case study. Overall, the integration of AI into dental diagnostics can enhance diagnostic accuracy and promote more cost-effective and patient-centric care.

Our appraisal of the literature does not come without limitations. While the case study illustrates AI’s potential to improve diagnostic accuracy and streamline treatment planning, it represents an illustrative example rather than a comprehensive validation of the accuracy of the technology. Additionally, the review conducted was narrative in nature, which limits the strength of the conclusions. Furthermore, while the Observational Diagnostics (ODs) framework offers a structured approach to training AI systems, the lack of direct comparison to large-scale, randomised clinical studies means that its clinical applicability remains preliminary. This highlights the need for further refinement and validation to support a broader implementation in complex dental treatments.

## 7. Conclusions

AI-driven tools have become indispensable in handling vast databases with remarkable speed, improving the efficiency and accuracy of clinical decisions. These tools excel in rapidly analysing large volumes of medical data, identifying intricate patterns in imaging, and enhancing diagnostic precision, especially in detecting subtle anomalies. Unlike human clinicians, AI is not subject to fatigue, ensuring consistent, standardised results across a wide range of cases, which helps minimise human error. While AI does not replace human expertise, it complements it by fostering a collaborative diagnostic process, allowing healthcare professionals to focus on more complex aspects of patient care [16,17,18,19].

In dental practice, AI systems’ ability to generate comprehensive diagnostic reports and treatment plans in real-time creates a collaborative environment between the dentist and technology. The case study presented here showcases how AI has become a powerful tool to enhance clinical decision-making by offering immediate feedback on missed diagnoses or unnecessary treatments. In particular, the integration of ODs as foundational elements helps in developing AI systems that adhere to evidence-based diagnostic and treatment guidelines. Such AI systems hold the potential to improve diagnostic accuracy, reduce variability, and further elevate clinical decision-making. Looking ahead, AI has the potential to replicate the clinical decision-making of a trained dentist, not only complementing human expertise but also elevating the overall standard of care for patients.

## Figures and Tables

**Table 1:** Example of studies assessing AI-powered diagnosis of dental pathologies [10,11,12,13,14,15,16,17,18,19,20,21,22]. PPV, positive predictive value; NPV, negative predictive value; Acc, accuracy; mIoU, mean Intersection over Union; AUC, Area Under the Receiver Operating Characteristic Curve.

Reference	Dental Condition	Sub-Category	Image Source	Results
Li W. et al. [10]	Periodontitis	Gingivitis	Digital Photographs	Sensitivity: 0.75, Specificity: 0.73, Accuracy: 0.74, Precision: 0.74
Lin PL et al. [11]		Alveolar Bone Loss	Periapical Radiographs	Sensitivity (True Positive Fraction): 0.925, Specificity (True Negative Fraction): 0.86
Lee CT. et al. [12]		Alveolar Bone Loss	Periapical Radiographs	Sensitivity: 0.80, Specificity: 0.99, Accuracy: 0.99, AUC: 0.98
Krois et al. [13]		Alveolar Bone Loss	Panoramic Radiographs	Sensitivity: 0.72, Specificity: 0.83, Accuracy: 0.81
M. B. H. et al. [14]		Periodontal Bone Destruction	Periapical Radiographs	Sensitivity: 0.92, Specificity: 0.71, Accuracy: 0.81, NPV: 0.90, Precision: 0.76,
Khan et al. [15]		Bone Recession and Interradicular Radiolucency	Periapical Radiographs	mIoU: 0.501, Dice score: 0.569
Kim J et al. [16]		Alveolar Bone Loss	Panoramic Radiographs	Sensitivity: 0.77, Specificity: 0.95, PPV: 0.73, NPV: 0.96, AUC: 0.95, F1-score: 0.75
Ghaedi et al. [17]	Endodontics	Detect and Score Caries Lesions	Intraoral Optical Occlusal Tooth Surface Images	Sensitivity: 0.83, Specificity: 0.983, Accuracy: 0.863
Berdouses et al. [18]		Occlusal Caries Lesions	Photographic Coloured Images	Sensitivity: 0.80, Specificity: 0.74, Accuracy: 0.80, Precision: 0.86, Recall: 0.86, F1-score: 0.85, AUC: 0.98
Pauwels et al. [19]		Periapical Lesions	Intraoral Radiographs	Sensitivity: 0.79, Specificity: 0.88, AUC: 0.86
Ekert et al. [20]		Periapical Lesions	Panoramic Radiographs	Sensitivity: 0.65, Specificity: 0.87, AUC: 0.85, PPV: 0.49, NPV: 0.93
Bayraktar et al. [21]		Interproximal Caries Lesions	Bitewing Radiographs	Sensitivity: 0.722, Specificity: 0.981, Accuracy: 0.945, PPV: 0.865, NPV: 0.954, AUC: 0.871
Toshihito et al. [22]	Prosthodontics	Partial Edentulous Arches	Oral Photographs	Maxilla: Sensitivity: 1.00, Accuracy: 0.995, Precision: 0.25, AUC: 0.99, Mandible Sensitivity: 1.00, Accuracy: 0.997, Precision: 0.25, AUC: 0.98

**Table 2:** Example of studies assessing AI-powered treatment planning.

References	Specialty	Sub-Category	Image Source	Conclusion
Bonfanti et al. [40]	Endodontics	Endodontically Treated Teeth	OPG	Treatment decisions using orthopantomography can be improved by using AI
Lee et al. [41]	Orthodontics	Orthognathic Surgery	Cephalograms	Indications of orthognathic surgery and orthodontic treatment based on images showed a significant success rate
Suhail et al. [42]	Orthodontics	Decision-making for teeth extraction	Patient records including facial photographs	AI was helpful in extraction and treatment planning

AI: Artificial Intelligence, OPG: Orthopantomograph (Orthopantomogram).

**Table 3:** Dental caries detection (conventional method vs. AI-based ODs and treatment plan).

	Tooth, Surface	Detection Mode	Findings/OD	Treatment Plan (Procedure)	Item Code	Cost	Inference
Dentist	23	Inspection	Initial caries	Adhesive restoration—anterior tooth—direct	522	$228.99	Dentist false positive
	14	Inspection	Moderate caries	Adhesive restoration—posterior tooth—direct	533	$286.01	Dentist true positive
CoTreat Navigator^®^	36D ^4^	PBW ^1^	Initial Stage ICCMS RA 3 ^2^	Adhesive restoration—posterior tooth—direct	532–535	$244.53–$383.93	Dentist false negative
	36B	Photo	Moderate caries ICDAS Code 3 ^3^				
	14DM	PBW ^1^	Initial Stage ICCMS RA 3 ^2^	Adhesive restoration—posterior tooth—direct	533–535	$286.01–$383.93	Dentist true positive
	14B	Photo	Moderate caries ICDAS Code 3 ^3^				

^1^ PBW = Posterior Bitewing (Intra-oral radiograph), ^2^ RA 3 = Primary Caries (Virgin Tooth) Radiolucency—Initial Stage ICCMS RA 3—Radiolucency limited to outer 1/3rd of dentine, ^3^ Code 3 = Primary Caries (Virgin Tooth) Discoloration—Moderate caries ICDAS Code 3—Distinct loss of enamel integrity with no visible dentine, viewed from the occlusal, buccal or lingual direction, appears as discolouration on a wet surface. ^4^ The teeth are numbered using the Federation Dentaire Internationale (FDI) system—a two-digit system, the first digit indicates the quadrant (1 through 4 for permanent and 5 through 8 for deciduous teeth) and the second digit indicates the tooth type (1 through 8 for permanent or 1 through 5 for deciduous teeth). The tooth surfaces are represented by the alphabets—La, Li, B, P, D, M, O, and I, to indicate the Labial, Lingual, Buccal, Palatal, Distal, Mesial, Occlusal and Incisal surfaces, respectively.

## Data Availability

Data are presented in the manuscript; raw data are available upon reasonable request.

## Supplementary Materials

The following supporting information can be downloaded at: https://www.mdpi.com/article/10.3390/bioengineering12010009/s1, Table S1: CoTreat Caries ODs; Table S2: OD-based treatment guidelines Table S3: Detection of Other Dental Conditions/Diseases (Conventional Method v/s AI-based Observational Diagnostics and Treatment Plan), Figure S1: CoTreat Note.

## Appendix A

**Table A1:** Commercially available AI-powered Dentist assistance products (as accessed on 30 September 2024).

Company	Product	Features
CoTreat https://www.cotreat.ai	CoTreat	Automated analysis Anatomy annotation Caries detection Treatment plan suggestion Patient data analysis
Dental Monitoring https://dentalmonitoring.com/our-solutions/	DM intelligent platform	Orthodontics monitoring
Eyes of AI https://www.eyesofai.com/products/ai-pathologies-detection	EAI Pathology	Automated analysis Anatomy annotation Caries detection
Glidewell.io https://glidewell.io/fastdesignio-software/	CrownAI and MarginAI	Patient data analysis Crown preparation margin analysis
ORCA Dental AI https://orca-ai.com/solutions/	ORCA AI	Automated analysis Image enhancement Anatomy annotation Caries detection Orthodontic treatment planning
ORYX https://www.oryxdentalsoftware.com/oryxbot	Oryxbot	Automated analysis Clinical Charting Caries detection Patient data analysis Treatment plan suggestion
Overjet https://www.overjet.com/solutions/dentists	Overjet Clinical Intelligence Platform	Automated analysis Anatomy annotation Caries detection
Pearl https://www.hellopearl.com/products/second-opinion	Second Opionion	Automated analysis Anatomy annotation Caries detection Treatment plan suggestion Patient data analysis
Smilecloud https://www.smilecloud.com/#cloud	Smilecloud	Digital smile design
VideaHealth https://www.videa.ai	VideaAI	Automated analysis Caries detection Treatment plan suggestion
