# Supplementary material for: Observational Diagnostics: The Building Block of AI-Powered Visual Aid for Dental Practitioners
Source: Bioengineering (Basel). 2024 Dec 25;12(1):9. doi: 10.3390/bioengineering12010009 (PMC11759822; doi:10.3390/bioengineering12010009)

Supplementary Files

Supplementary Table S1: CoTreat Caries ODs [36,37]

Category	Sub-category	Observation name
Caries	Radiolucency	Initial Stage ICCMS RA 1 - Radiolucency in outer 1/2 of enamel
Caries	Radiolucency	Initial Stage ICCMS RA 2 - Radiolucency in inner 1/2 of enamel +/- DEJ
Caries	Radiolucency	Initial Stage ICCMS RA 3 - Radiolucency limited to outer 1/3rd of dentine
Caries	Radiolucency	Moderate Stage ICCMS RB 4 - Radiolucency limited to middle 1/3rd of dentine
Caries	Radiolucency	Extensive Stage ICCMS RC 5 - Radiolucency in inner 1/3rd of dentine
Caries	Radiolucency	Extensive Stage ICCMS RC 6 - Radiolucency into pulp
Caries	Discolouration	Initial Caries ICDAS Code 2 - Non-cavitated brown carious discolouration or shadow confined to the enamel on a wet surface
Caries	Discolouration	Moderate caries ICDAS Code 3 - Distinct loss of enamel integrity with no visible dentine, viewed from the occlusal, buccal or lingual direction, appears as discolouration on a wet surface
Caries	Discolouration	Moderate caries ICDAS Code 4 - Grey, blue, brown, dentine shadow visible on the marginal ridge or dark shadow from dentine with or without localised enamel breakdown
Caries	Cavity	Extensive Caries ICDAS Code 5 - Less than half the surface is carious, distinct cavity with opaque or discoloured enamel with visible dentine
Caries	Cavity	Extensive Caries ICDAS Code 6 - More than half the surface is a carious, distinct cavity with visible dentine
Caries	Root Caries	Root Caries Code 1 - Initial lesion - There is a clearly demarcated area on the root surface (cementum) or at the CEJ that is discoloured (light/dark brown, black) without cavitation and loss of anatomical contour < 0.5 mm
Caries	Root Caries	Root Caries Code 2 - Moderate lesion - A clearly demarcated area on the root surface or at the CEJ that is discoloured (light/dark brown, black) with cavitation and loss of anatomical contour ≥ 0.5 mm < 2 mm present
Caries	Root Caries	Root Caries Code 2 - Extensive lesion - A clearly demarcated area on the root surface (cementum) or at the CEJ that is discoloured (light/dark brown, black) with cavitation and loss of anatomical contour > 2 mm present
Caries	Root Caries	Radiolucency indicative of root caries
Caries	Secondary Caries	CARS - ICDAS 2 - Distinct visual change in enamel/dentine adjacent to a restoration/sealant margin
Caries	Secondary Caries	CARS - ICDAS 3 - Cavitation at the margin of the restoration/sealant less than 0.5mm, in addition to either opacity or discolouration

		consistent with demineralisation
Caries	Secondary Caries	CARS - ICDAS 4 - Marginal caries in enamel/dentine/cementum adjacent to restoration/sealant with underlying dark shadow from dentine
Caries	Secondary Caries	CARS - ICDAS 5 - Distinct cavity adjacent to restoration/sealant with visible dentine in the interfacial space with a gap of > 0.5 mm in width
Caries	Secondary Caries	CARS - ICDAS 6 - Extensive distinct cavity adjacent to restoration/sealant with visible dentine in the interfacial space with a gap of > 0.5 mm in width. The cavity may be deep or wide on both walls and at the base

CEJ: cementoenamel junction, ICCMS: International Caries Classification and Management System, ICDAS: International Caries Detection and Assessment System.

Supplementary Table S2: OD-based general treatment guidelines [36].

<table>
<tr><th>Category</th><th>Subcategory</th><th>OD text</th><th>Treatment Guidelines*</th></tr>
<tr><td rowspan="2">Caries</td><td>Radiolucency</td><td>Initial caries:

A. ICCMS RA 1 - radiolucency in outer 1/2 of enamel

B. Initial stage ICCMS RA 2 - radiolucency in inner 1/2 of enamel +/- DEJ</td><td rowspan="2">Inactive: No lesion-specific treatment

Active: Non-Operative Care

1. Oral Hygiene instructions A. Tooth brushing 2/day with fluoride toothpaste (≥ 1,000 ppm F), following the dental team instructions. B. Mechanical removal of biofilm.

2. Clinically applied topical fluoride (all surfaces).

3. Pit and Fissure Caries: Resin-based sealants, Glass ionomer sealants.

4. Proximal Caries: Resin-based sealants/infiltrants.</td></tr>
<tr><td>Discolouration</td><td>Initial caries

ICDAS Code 2 - non-cavitated brown carious discolouration or shadow confined to the enamel on a wet surface</td></tr>
</table>

DEJ: Dentino-enamel junction, ICDAS: International Caries Detection and Assessment System, OD: Observational Diagnostics, , ICCMS: International Caries Classification and Management System. *Note that individual operative procedures and contraindications are not reported in this table.

Supplementary Table S3: Detection of Other Dental Conditions/Diseases (Conventional Method v/s AI-based Observational Diagnostics and Treatment Plan) [47]

	Tooth, Surface	Detection Mode	Findings/OD	Treatment plan (Procedure)	Item code
**Dentist**	16	PBW[1]	Severe bone loss	Active non-surgical periodontal therapy – per quadrant	250
	47	PBW[1]	Severe bone loss	Active non-surgical periodontal therapy – per quadrant	250
	17	OPG[2]	Missing	Review after periodontal therapy	NA
	26, 46	Inspection	Root pieces	Extraction	311
	31, 41	Inspection	Calculus	Included in periodontal therapy	250
	31, 41	Inspection	Recession	Review after periodontal therapy	NA
	31, 41	Inspection	Moderate Gingivitis	Included in periodontal therapy	250
	31, 41	Inspection	Spacing	Review after periodontal therapy	NA
	38, 48	OPG[2]	Impacted	Review after periodontal therapy	NA
**CoTreat (Navigator)**	16	Photo	Alveolar Bone Loss - Stage II[3]	Periodontal debridement – per tooth	222
	37, 27DM, 47DM	PBW[1]	Alveolar Bone Loss - Stage III[4]	Active non-surgical periodontal therapy – per quadrant	250
	31, 32, 41, 42	Photo	Supragingival calculus[5]	Included in Active non-surgical periodontal therapy – per quadrant	Included in Item Code 250
	16D ...	PBW[1]	Radiopaque calculus[6]	Included in Active non-surgical periodontal therapy – per quadrant	Included in Item Code 250
	11, 21	Photo	Tooth Wear - Mild[7]	Adhesive restoration – one surface – anterior tooth – direct	521
	31B, 41B	Photo	Gum Recession - Millers Class III[8]	Review after periodontal therapy	NA
	26, 46	Photo	Retained Roots[9]	Extraction	311

	11, 12, 21, 31, 32, 41, 42	Photo	Malocclusion - Spacing[10]	Review after periodontal therapy	NA
	38, 48	OPG[2]	Wisdom Teeth - Fully Impacted[11]	Review after periodontal therapy	NA
	17	OPG[2]	Restorable Edentulous Space[12]	Review after periodontal therapy	NA
	31La, 41La, 42La	Photo	Gingivitis Grade 3[13]	Included in Active non-surgical periodontal therapy – per quadrant	Included in Item Code 250

1. PBW = Posterior Bitewing (Intra-oral radiograph)
2. OPG = Orthopantomograph (Orthopantomogram)
3. Alveolar Bone Loss - Stage II - 15-33% bone loss confined to the coronal third, mostly horizontal
4. Alveolar Bone Loss - Stage III - Horizontal bone loss extends to middle or apical third, and/or vertical bone loss of ≥3mm, and furcation radiolucency, ≤ 4 teeth missing
5. Dental Calculus - Presence of supragingival calculus
6. Dental Calculus - Radiopaque calculus - Radiopaque irregularity at cementoenamel junction or extending beyond or superimposed over the root surface contour Teeth #16D, 27D, 37D, 33M, 32MD, 31MD, 41MD, 42MD, 43D, 45M,
7. Tooth Wear - Mild or moderate Score 1 - Mild - Wear limited to enamel or Tooth Surface Loss of ≤25%
8. Gum Recession - Millers Class III Marginal tissue recession, extending up to or beyond MGJ, with interdental bone loss and soft tissue loss on buccal or labial surface
9. Retained Roots in Soft Tissue - Submerged root fragments with no periapical radiolucency
10. Malocclusion - Spacing
11. Wisdom Teeth - Fully Impacted but in close relation to IAC/IAN (Interrupted white line or narrow canal or dark/bent roots visible)

12. Restorable Edentulous Space - Missing Tooth per arch - Kennedy Class II = Unilateral edentulous area located posterior to the remaining natural teeth
13. Gingivitis Grade 3 = Moderate inflammation, marked redness, oedema and/or hypertrophy of marginal or papillary gingiva

Supplementary Figure S1: CoTreat Note [47]. CoTreat automatically generates a comprehensive Observational Diagnostics Report (Navigator Report) and Treatment Plan. CoTreat (Colleague Note) alerts the dentist on missed findings (false negatives) and false positives (to reduce unnecessary harm and costs to patients), if any.

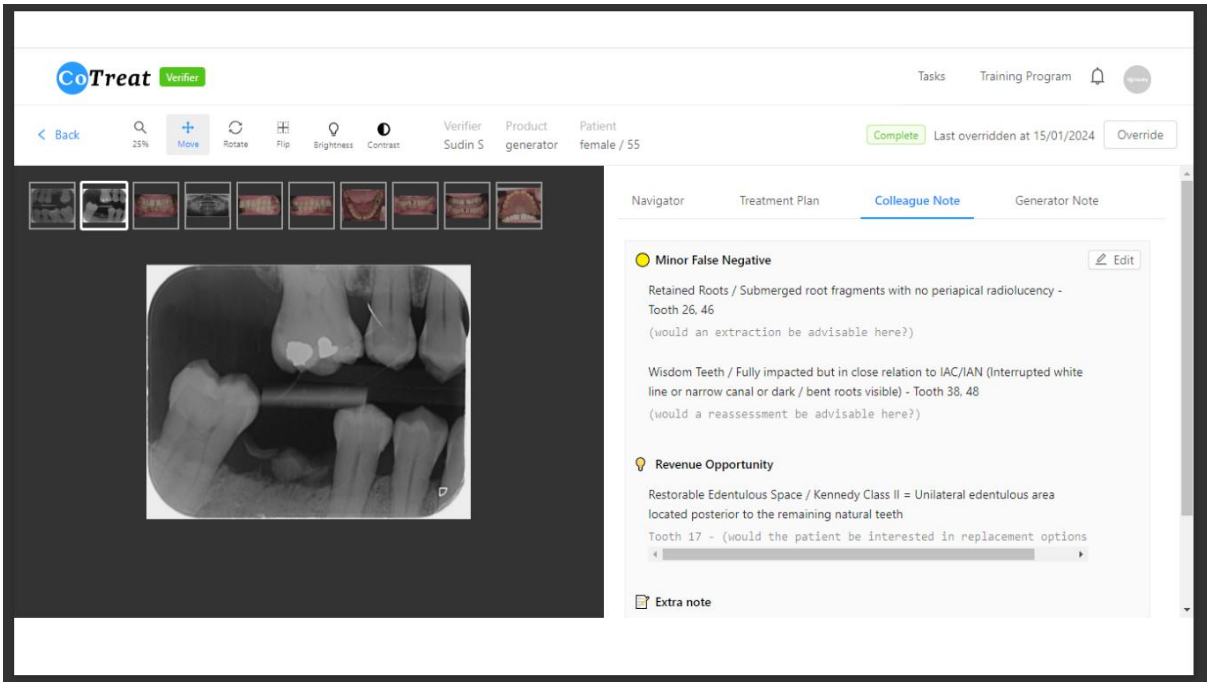

Supplement: Supplementary file 1 [file bioengineering-12-00009-s001.zip › bioengineering-3308205-supplementary.pdf]
